# Moral Distress Trajectories of Physicians 1 Year after the COVID-19 Outbreak: A Grounded Theory Study

**DOI:** 10.3390/ijerph182413367

**Published:** 2021-12-19

**Authors:** Giulia Lamiani, Davide Biscardi, Elaine C. Meyer, Alberto Giannini, Elena Vegni

**Affiliations:** 1 Department of Health Sciences, Università degli Studi di Milano, 20142 Milan, Italy; elena.vegni@unimi.it; 2 Unit of Clinical Psychology, Santi Paolo e Carlo Hospital, 20142 Milan, Italy; davide.biscardi@asst-santipaolocarlo.it; 3 Center for Bioethics, Harvard Medical School, Boston, MA 02115, USA; elainecmeyer@gmail.com; 4 Boston Children’s Hospital, Harvard Medical School, Boston, MA 02115, USA; 5 Pediatric Intensive Care Unit, Spedali Civili di Brescia Hospital, 25123 Brescia, Italy; alberto.giannini@asst-spedalicivili.it

**Keywords:** COVID-19, psychological stress, ethics, moral distress, intensive care, emergency medicine, grounded theory

## Abstract

The COVID-19 pandemic has confronted emergency and critical care physicians with unprecedented ethically challenging situations. The aim of this paper was to explore physicians’ experience of moral distress during the pandemic. A qualitative multicenter study was conducted using grounded theory. We recruited 15 emergency and critical care physicians who worked in six hospitals from the Lombardy region of Italy. Semi-structured interviews about their professional experience of moral distress were conducted from November 2020–February 2021 (1 year after the pandemic outbreak). The transcripts were qualitatively analyzed following open, axial, and selective coding. A model of moral distress was generated around the core category of Being a Good Doctor. Several Pandemic Stressors threatened the sense of Being a Good Doctor, causing moral distress. Pandemic Stressors included limited healthcare resources, intensified patient triage, changeable selection criteria, limited therapeutic/clinical knowledge, and patient isolation. Emotions of Moral Distress included powerlessness, frustration/anger, and sadness. Physicians presented different Individual Responses to cope with moral distress, such as avoidance, acquiescence, reinterpretation, and resistance. These Individual Responses generated different Moral Outcomes, such as moral residue, disengagement, or moral integrity. The Working Environment, especially the team and organizational culture, was instrumental in restoring or disrupting moral integrity. In order for physicians to manage moral distress successfully, it was important to use reinterpretation, that is, to find new ways of enacting their own values by reframing morally distressing situations, and to perceive a cooperative and supportive Working Environment.

## 1. Introduction

The COVID-19 epidemic was declared by the World Health Organization [1] as a pandemic on 11 March 2020. Italy was the first European country to be affected following the outbreak in China. The rapid and unexpected evolution of the pandemic generated enormous pressure on the Italian healthcare system [2]. The increasing number of patients, and the limited clinical supplies and equipment compared to the mounting healthcare needs, as well as the paucity of knowledge about the disease overwhelmed the healthcare system [3,4]. The imbalance between the healthcare needs and the available resources forced physicians to grapple with extremely difficult and unprecedented clinical choices [5]. Under these dramatic circumstances, the main guiding ethical principles—respect for autonomy, beneficence, non-maleficence, and distributive justice- were continuously utilized to guide decisions that balanced the benefits and harms of individual patients with that of the larger community [2]. Concomitantly, the decision to prohibit family visitation, in order to contain the spread of the virus, further amplified the burden on healthcare professionals when caring for huge numbers of dying patients, without the customary comfort of their loved ones [6].

The circumstances of the pandemic had a strong impact on the emotional well-being of healthcare professionals, causing anxiety, depression, post-traumatic stress, and insomnia [7,8]. Some authors suggest these situations may have also precipitated moral distress, a distinct type of stress associated with the moral dimension of being healthcare professionals [9,10].

Moral distress was originally conceptualized by Jameton as the painful feeling that occurs when “one knows the right thing to do, but institutional constraints make it nearly impossible to purse the right course of action” [11] (p. 6). Since this first definition, other authors contributed to refine the concept of moral distress by adopting broader definitions. Recently, Batho, and Pitton [12] argued that the main characteristic of moral distress is the perception of being morally compromised for not being able to be oneself in a situation in which you feel that you should (but were not) able to do the right thing. Epstein and Hamric proposed a crescendo effect model of moral distress [13] by focusing on the difference between the initial moral distress that is experienced during a situation, and the moral residue that represents the lingering angst that continues after the event. Webster and Bayliss defined moral residue as “that which each of us carries with us from those times in our lives when in the face of moral distress we have seriously compromised ourselves or allowed ourselves to be compromised” [14] (p. 208). This residual distress can cause damage over time, especially when healthcare professionals are repeatedly exposed to morally distressing events, and do not succeed in restoring moral integrity [14]. Several studies showed that clinicians’ moral distress in the ICU is positively associated with burnout, depression, withdrawal from patient care, and job resignation [15,16,17,18].

The pandemic, as an unprecedented and exceptional global health crisis, may have enhanced the moral distress experience of intensive and emergency care physicians, particularly among those who had triage responsibility, and were closely involved in the acute care of COVID-19 patients [9]. However, to our knowledge, no study has explored this experience so far.

The aim of this research was to explore the experience of moral distress among emergency and intensive care physicians one year after the pandemic outbreak.

## 2. Materials and Methods

### 2.1. Study Design

The study was designed according to the principles of grounded theory, which is a qualitative, inductive methodology originally developed by Glaser and Strauss [19] to study psychosocial processes, and to generate theory originating from the data. The study incorporated a multicenter design using in-depth semi-structured interviews.

### 2.2. Participants’ Recruiting

Emergency and intensive care physicians were purposefully selected from the intensive care units (ICU) and emergency departments (ED) of six hospitals located in Lombardy, which was the epicenter of the pandemic in Italy. Participants were selected if they worked in ED or ICU settings, had at least 5 years of experience as a physician, and provided direct care to COVID-19 patients. The chief physicians of the aforementioned units proposed the study to physician staff members who met the eligibility criteria. Phone numbers of interested physicians were provided to the principal investigator (GL), who then called prospective participants to explain the study and, if interested, to schedule an interview. According to the principles of theoretical sampling [20], participants were progressively recruited with the aim of ensuring variability in gender, triage experience, responsibility and role, and self-reported moral distress levels. Physicians who agreed to participate completed a baseline sociodemographic questionnaire online, and the Moral Distress Thermometer (MDT) [21], a 1-item measure ranging from 0 (none) to 10 (worst possible). In addition, a snowball technique of recruiting was employed to identify other physicians who might be willing to participate in the study. As data collection and analysis were conducted simultaneously, we stopped recruiting when we reached data saturation, that is, when no new categories emerged from the analysis of the interviews.

### 2.3. Data Collection

The interviews were conducted online between November 2020 and February 2021 (one year after the pandemic outbreak) by two researchers (GL; DB) whose backgrounds are in clinical psychology and psychotherapy, and who had previous experience conducting in depth-interviews [22]. The interviews followed a semi-structured, conversational style. At the beginning, participants were offered a brief definition of moral distress based on the work of Jameton [11], and were asked whether the experience of moral distress resonated with them during the COVID-19 pandemic. This question was followed by other questions formulated in advance (see Table 1). During the interviews, the researchers followed the flow of the interviewees, and facilitated the expression of the experience by using active listening, reformulation, and checking techniques [23].

### 2.4. Data Analysis

Sociodemographic data were analyzed using descriptive statistics. The interviews were audio-recorded, transcribed verbatim, and analyzed according to the principles of grounded theory by the same researchers who conducted the interviews. The analysis followed three steps: open, axial, and selective coding [24]. The open coding aimed to freely identify codes that described the concepts expressed in the interviews, and was conducted independently by the researchers. In the axial coding, the researchers met several times to share the codes identified, and aggregate them progressively into broader categories. In this phase, researchers discussed the emerging categories, and aimed to determine the relationships and logical connections between them, using notes and graphical diagrams. During the selective coding (the final and most abstract step of analysis), researchers aimed to identify the core category that is considered the pivotal concept in grounded theory that explains the whole process under investigation, and connects all the other categories that have been identified. The core category ultimately represents the central thesis of the research [25]. In this stage of analysis, a third researcher (EV), whose background is in psychology, joined the meetings to provide an external perspective on the core category and its articulation with the other categories. Once consensus was reached, and the model of the moral distress experience was defined and graphically depicted, a meeting with co-authors, an ICU physician (AG), and a nurse/psychologist/bioethicist (ECM) was held to receive feedback on the fit, relevance, and coherence of the model identified.

## 3. Results

### 3.1. Participants

Of the 18 physicians contacted, 15 (83%) physicians were interviewed (5 ED, 10 ICU). The interviews lasted on average 51.82 min (range 32–60). Nine physicians (60%) were female. The mean age was 46 (SD = 5.24), and the mean years of experience was 18 (SD = 6.14). Ten (67%) were responsible for patient triage during the pandemic. The sample’s description is provided in Table 2.

### 3.2. Model of the Moral Distress Process

**Figure 1:**
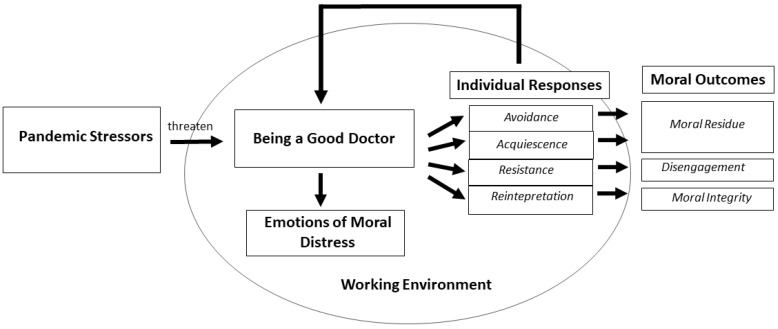
Model of the moral distress process during COVID-19 pandemic.

The experience of moral distress was characterized by a process that unfolded around the core category of Being a Good Doctor in the face of the pandemic (Figure 1).

Being a Good Doctor meant different things for different physicians: to cure patients, to be informed and/or prepared, to guide relationships with families. Regardless of what each participant meant for being a good professional, the Pandemic Stressors represented challenges for all. Pandemic Stressors included limited healthcare resources, intensified patient triage, changeable selection criteria, limited therapeutic/clinical knowledge, and patient isolation. Pandemic Stressors threatened the sense of Being a Good Doctor, generating moral distress. Emotions of Moral Distress were powerlessness, frustration, anger, and sadness. Physicians described different Individual Responses to moral distress, such as avoidance, acquiescence, resistance, and reinterpretation. These responses may or may have not succeeded in restoring the sense of Being a Good Doctor, and therefore generated different Moral Outcomes, such as moral residue, disengagement, or moral integrity. In addition to Individual Responses, the Working Environment, especially the team and perceived organizational culture, was instrumental in influencing the Moral Outcomes. In the sections below, the categories of the model are described, and illustrative quotes are reported from the interviews. The quotes are followed by the participant number.

#### 3.2.1. Pandemic Stressors

- Limited Healthcare Resources. One of the main factors that caused moral distress was the experience that resources were woefully inadequate: not enough oxygen, not enough ICU beds, not enough physicians. The stressful experience of working with limited resources was a difficult shift, and fostered the sense of not providing good care. “*We didn’t have any more beds in the ICU, we found ourselves distributing opioid to do palliative care to people who normally we would have saved*”. #12.
- Intensified Patient Triage. In the context of limited healthcare resources, physicians were confronted with the struggle of triaging patients. Although the experience of triage was familiar to many, it was extraordinarily difficult because of the sheer volume of consequential decisions to make, and triage criteria that differed from pre-pandemic standards. “*There was a time during which we assessed nearly 250 patients a day…We lowered the standard of care at that point and that was difficult for me to bear*.” #6. “*I felt like a judge who should play God and say: «You live, you die». I became like a robot to survive*.” #2.
- Changeable Selection Criteria. Another distressing factor was the lack of consistency of the selection criteria due to the rapid changes in knowledge about the disease, or to the different interpretation of selection criteria within the team. The changeable selection criteria led to a deep sense of injustice. “*For me the lack of fairness was unjustifiable because every day they changed the cards on the table. If you had a particularly wired colleague (on the shift before you) who occupied all the beds and ventilators, then you had nothing left for the other patients. There are rules that apply to everyone. Otherwise, it’s all random!*” #9.
- Limited Therapeutic/Clinical Knowledge. Many physicians reported that acting in the context of limited knowledge of the disease and therapeutics was a source of moral distress. Oftentimes the limited clinical knowledge led to the use of drugs and/or procedures without sufficient clinical evidence, thereby risking damage to patients. “*Sometimes, we do damage that is irreversible because there is still no guideline, there is no standardized procedure, there is no blood gas reference to say now you better intubate, now no*.” #14.
- Patient Isolation. Another factor triggering moral distress was patient isolation and the separation from family members during such meaningful moments as during the end-of-life. Many physicians found themselves as witness to patients’ loneliness, and thrust into intimate moments at the end-of-life, further amplifying moral distress. “*I remember a patient with whom we were doing palliation. One evening, at the end of my shift, I saw him looking at a yellow frame containing photos of his children and grandchildren. He was crying, aware that he would die. He died the next day and only I witnessed this scene. I still carry the scene with me like a flash of sadness. Maybe we could have done more to connect him with his family, other than video calls*.” #6.

#### 3.2.2. Emotions of Moral Distress

- Powerlessness and Inadequacy. The predominant feeling related to the experience of moral distress was a deep sense of powerlessness to provide care that could be considered as adequate. “*Our weapons par excellence are the endotracheal tubes and the ventilators. We used them but it was like water for the patients. It didn’t change anything. I remember sudden deaths without the possibility of doing anything. It’s the powerlessness that comes to the surface*.” #14.
- Frustration and Anger. The impossibility of upholding the standard of care or acting according to one’s conscience was often followed by frustration and anger. “*Every day we asked for supplies. We asked for anything in order not to see people die, but there were no resources! So, I was a frustrated person*.” #4.
- Sadness. Many physicians described sadness when witnessing overwhelming situations where they felt impotent to fulfill their vision of what it meant to Be a Good Doctor. Sadness emerged as an intimate response when bearing witness to human suffering on such a massive scale. “*During the very first days…I heard only the sound of sirens, the beds seemed to be never enough, the patients were going very bad, they dropped like flies. Those days I said crying: «It will be a massacre!» I really had to say that I was suffering for humankind*.” #1.

#### 3.2.3. Individual Responses

- Avoidance. Some physicians tried to protect themselves by avoiding exposure to morally stressful conditions or tasks, such as communicating with patients’ family members by phone. Others, especially during triage, avoided human contact with critical patients to mitigate moral distress. “*I was so exhausted that I didn’t want to look patients in the face anymore. I could not make it to see the umpteenth patient who did not breathe. For this reason, I used to take the patient’s chart…and I would say: «This one is to be intubated, this one is not»*.” #2.
- Acquiescence. Another response to moral distress was to passively follow the orders, and accept the realities without challenging them. Faced with the incongruence between their own values and the grim reality, some physicians silenced and disregarded their professional values in order to accommodate with the situation. “*I remember a recent young patient, who arrived in the ED, suffering from a tumor with a poor prognosis. He was probably also affected by COVID because he had the characteristic symptoms, but my colleagues did not want to test him for COVID to discharge him quickly and prevent him from occupying an additional bed, given the poor prognosis. I felt very guilty towards the patient and his family*.” #3.
- Resistance. Another possible response was proactive resistance to situations or orders that were perceived as morally wrong. Confronted with the incongruence between one’s own values and the desperate reality, some physicians spoke up to their superiors, and further expressed concern about morally questionable practices or behaviors. Some issued formal complaints to political institutions, and/or proposed new healthcare procedures, whereas others, in specific situations, disregarded certain rules perceived as unethical. “*Unfortunately, the approach was “We are at war. There are generals and there are soldiers: soldiers obey what the generals say. Therefore, it was necessary to carry out even improper orders that had to do with the death of people. I had several fights due to the fact that I just couldn’t handle some of the orders*.” #8.
- Reinterpretation. Another possible response among physicians was to reinterpret their role as a Good Doctor by reframing the reality, recognizing the exceptional circumstances, and engaging their creativity to achieve different ways to care for patients. This creative, adaptive process required deep emotional labor. “*In the first week when I put the patients naked in a black sack, I suffered, and I asked myself “With my being a doctor, what can I preserve and what might I be able to change?” And from there, I started to write my name on the gown, so that the patients knew who I was, to take care of the daily phone calls to relatives… and to fight at the Ministry of Health so that the deceased could be put dressed in the sacks. I prayed for them while we closed the sack. Well, I don’t know if these things helped the patients, but they helped me! I started to regain my sense of purpose again with the things I believed in with all of myself*.” #1.

#### 3.2.4. Working Environment

- Team Culture. The team culture to which physicians belonged served to modulate moral distress. For some, a supportive team, which was open to discussion, permitted physicians not to feel abandoned while struggling with moral distress. “*I worked in a very organized structure with a group of culturally prepared people, some of whom are old friends. I believe that having the technical and human resources (available), helped me not to feel inadequate*.” #1. On the contrary, physicians who worked within less supportive team cultures reported a sense of loneliness and responsibility that amplified moral distress.
- Organizational Culture. Organizational culture also played a prominent role in modulating physicians’ moral distress. Physicians who perceived their organization as supportive, oriented to listening, and motivated to respond to the problems arising during the pandemic reported diminished moral distress. On the contrary, physicians who perceived their organization as repressive, “military oriented”, and unresponsive to suggestions reported an exacerbation of the moral distress emotional experience, which easily led to disengagement. “*You work a lot, but you think little. And this thing here during the pandemic has become devastating. The mantra was: «You don’t have to think. Act because I think for you.» But when in complex situations you stop thinking then you do atrocious things*.”#8.

#### 3.2.5. Moral Outcomes

- Moral Residue. Some physicians, especially those who utilized avoidance or acquiescence in response to moral distress, and who experienced a hierarchical, individualistic, or rigid work environment, reported lingering feelings of shame, regret, self-doubt, and guilt for not being able to act differently to effect change as if they should have been able to do so. “*I felt dirty, ugly inside having to say “No, there are no beds”. I would go home and cry. I had come to not want to see my daughter anymore. I didn’t want to see my face next to hers in the video call. I felt like I was dirtying her*.” #9.
- Disengagement. Some physicians reported feelings of loneliness, resignation, disillusionment, and lack of motivation, especially those who used resistance as the main response to moral distress, and who perceived their Working Environment as hierarchical, individualistic, and unresponsive to their suggestions. As a result, some physicians felt emotionally disengaged from the hospital, with a withdrawal in energy and a diminished sense of belonging. Disengagement was followed by the desire to change positions, or to leave the hospital and the profession altogether. “*The long wake of this situation was a detachment from the hospital management, a divestment, a disillusionment. Before COVID the hospital was like my home, now it’s a rented house*.” #12.
- Moral integrity. Some physicians, especially those who used reinterpretation in response to moral distress, and who perceived the Working Environment as participatory, collaborative, and receptive to suggestions, reported moral integrity and a restored sense of Being a Good Doctor. They described having been challenged, but emerging with a feeling of integrity, wholeness, and peace of conscience. “*We don’t always manage to cure but we can always care for. This helped me to preserve my wholeness during the pandemic because even on the worst days it can be honorable…knowing that you have done things right*.” #1.

## 4. Discussion

This qualitative study examined the experience of moral distress among emergency and critical care physicians during the COVID-19 pandemic in Italy. The grounded theory methodology allowed for capturing the process of moral distress: the causes, the related emotions, the responses used by physicians to face it, and its outcomes.

The most common causes of moral distress during the pandemic were related to situations in which physicians did not have enough resources to care for all incoming patients, or implemented medical treatment without a consolidated medical knowledge. Witnessing patients’ isolation and separation from family members was also source of deep moral distress [26]. The fact that these situations generated moral distress is confirmed by the many efforts put in the publication of recommendations about the allocation of intensive care treatments [2] and the communication with families living in complete isolation [27]. Despite some similarities with moral distress causes identified before COVID-19 [28,29,30], the causes identified in this study were specific to the pandemic context [31]. We know that moral distress may also arise in ordinary times from very different conditions and situations. Therefore, we argue that future literature on moral distress should focus less on causes, and more on the dynamics that may foster or reduce it [32]. By unveiling the moral distress process, this study offered new insights into individual and organizational responses that could influence its outcomes.

The pandemic stressors deeply threatened the physicians’ sense of being a good doctor, and therefore generated moral distress. As a result, many physicians reported feelings of powerlessness, frustration, anger, and sadness. These emotions are previously known correlates of moral distress [16,29,32]. However, our findings showed that the negative outcomes deriving from moral distress were not a destiny, but depended on the dynamic relationship between individual responses and the working environment [33,34].

Confronted with the painful pandemic-related moral distress, physicians spontaneously adopted different responses, some of which appeared more adaptive to restore a sense of being a good doctor. Consistent with the findings of Batho and Pitton [12], we found that physicians adopted mainly four responses to manage moral distress: avoidance, acquiescence, resistance, and reinterpretation. Avoidance of morally distressing situations emerged as an initial and provisional self-protective response. Acquiescence and resistance, on the contrary, were responses that engendered an internal struggle between silencing personal values and accepting the situation, or upholding personal values and resisting the status quo [12]. Both responses exacted a cost for physicians: either one of self-blame, guilt, or shame for not being able to stand up for what was right, and feeling morally compromised (in case of acquiescence), or one of loneliness and disengagement from the organization (in case of resistance). In other words, these strategies seem to pave the way to moral residue or disengagement, which have been previously reported as possible outcomes of unresolved moral distress [28,33]. Reinterpretation was the only response that allowed physicians to restore a sense of moral integrity by recognizing their limited degree of freedom in the situations, and finding new possible ways to be good doctors under challenging circumstances. Reinterpretation permitted one to uphold values by rediscovering new feasible ways of moral expression thanks to a reappraisal of the situation.

In addition to individual responses, the working environment, specifically the team and the organizational culture, played an important role in managing moral distress. It is becoming increasingly clear that outcomes associated with moral distress do not depend solely on individual characteristics, but also on the team functionality and ethical climate of the organization [34,35,36]. Some authors argue that the experience of moral distress may be an inevitable part of the work in the ICU environment, and that it is probably not even possible or beneficial to eradicate it altogether [29,37]. Indeed, moral distress may be considered a vital sign of our moral conscience [32], and an essential component of the caring profession that can act as a trigger for quality care improvement [38]. However, in order to leverage moral distress for organizational improvements, it is necessary that hospitals create spaces to listen to clinicians’ moral distress—as it already happens for incident reporting—or establish ethical rounds or moral case deliberation meetings to discuss the ethical aspects of care [39,40]. Our findings suggest that participatory, proactive, and attentive leadership; as well as teamwork that promotes the recognition, sharing, and reconciliation of emotional and ethical aspects of care among its members can help clinicians cope with moral distress.

The study has several limitations. As it involved a small convenience sample of physicians, the results are not generalizable. Future studies could investigate whether the proposed model of moral distress remains explicative in non-emergency/critical care contexts and among different professionals. Moreover, our experience with interviews leads us to think that some pandemic stressors also had a traumatic effect on physicians. Future research could explore the possible relationship between trauma and moral distress during the pandemic.

## 5. Conclusions

Our results demonstrate different trajectories of moral distress one year after the pandemic outbreak. The negative outcomes of moral distress, such as moral residue and disengagement, were influenced by a combination of individual responses and the working environment. The results suggest that in order for physicians to successfully manage moral distress during the pandemic, it is important to find new ways of enacting their own values by reframing morally distressing situations, and to perceive a cooperative and supportive working environment. In order to ensure a healthy emergency and intensive care workforce, and prevent job resignation, it will be important to promote a reinterpretation of morally distressing situations through ethical consultations and psychological interventions. In addition, healthcare organizations should promote positive teamwork and ethically supportive organizational cultures.

## Figures and Tables

**Table 1:** Interview guide.

Order	Questions
1	Moral distress has been defined as the distress experienced when you feel you cannot act according to what you think is correct/right in your profession. Does this experience resonate with you during this pandemic time in any way?
2	Do you recall a situation, since the beginning of the pandemic, where you think you experienced moral distress?
3	How did you feel in that situation?
4	What helped you to navigate/cope with that situation?

**Table 2:** Participants’ sociodemographic characteristics and moral distress levels.

Partecipant Code	Sex	Age	Experience (Years)	Work Setting during Pandemic	Role	Triage Responsibility (Yes/No)	Number of COVID-19 Patients Assisted	Moral Distress Score (0–10)
1	F	44	19	ICU	Attending	No	50–100	1
2	F	49	24	ED	Attending	Yes	50–100	7
3	F	32	6	ED	Consultant	No	>200	6
4	F	51	22	ICU and sub-intensive	Chief	Yes	>200	2
5	M	50	10	ICU	Attending	Yes	100–200	3
6	M	45	19	ICU	Attending	Yes	>200	5
7	M	48	23	ICU	Attending	Yes	100–200	1
8	M	42	15	ED	Attending	Yes	100–200	8
9	M	55	30	ICU	Chief	Yes	100–200	6
10	F	41	15	ED	Attending	No	>200	7
11	M	46	20	ICU	Attending	No	50–100	5
12	F	43	10	ICU and ALS vehicle	Attending	Yes	>200	8
13	F	46	18	ICU	Attending	No	>200	5
14	F	47	22	ED	Chief	Yes	>200	2
15	F	51	24	ICU	Attending	Yes	>200	3

## Data Availability

The data presented in this study are available in Italian on request from the corresponding author. The data are not publicly available due to confidentiality reasons.
